# Three New Lanostanoids from the Mushroom *Ganoderma tropicum*

**DOI:** 10.3390/molecules20023281

**Published:** 2015-02-16

**Authors:** Shuang-Shuang Zhang, Yu-Guang Wang, Qing-Yun Ma, Sheng-Zhuo Huang, Li-Li Hu, Hao-Fu Dai, Zhi-Fang Yu, You-Xing Zhao

**Affiliations:** 1 College of Food Science and Technology, Nanjing Agricultural University, Nanjing 210095, China; E-Mail: 2012208021@njau.edu.cn; 2 Key Laboratory of Biology and Genetic Resources of Tropical Crops, Institute of Tropical Bioscience and Biotechnology, Chinese Academy of Tropical Agricultural Sciences, Haikou 571101, China; E-Mails: wangyuguang@itbb.org.cn (Y.-G.W.); maqingyun@itbb.org.cn (Q.-Y.M.); huangshengzhuo@itbb.org.cn (S.-Z.H.); lili861025@aliyun.com (L.-L.H.); daihaofu@itbb.org.cn (H.-F.D.)

**Keywords:** Ganodermataceae, *Ganoderma tropicum*, lanostanoid triterpenes

## Abstract

Three new lanostanoid triterpenes—ganotropic acid (**1**), 3β,7β,15α,24-tetra-hydroxy-11,23-dioxo-lanost-8-en-26-oic acid (**2**) and 3β,7β,15α,28-tetrahydroxy-11,23-dioxo-lanost-8,16-dien-26-oic acid (**3**)—were isolated from the *n*-BuOH extract of the fruiting bodies of the mushroom *Ganoderma tropicum*. Their structures were elucidated by 1D and 2D NMR spectroscopy, as well as HR-EI-MS data.

## 1. Introduction

*Ganoderma*, the major genus in the family Ganodermataceae, are widely used to cure various chronic diseases such as hypertension, diabetes, hepatitis and cancers [1,2,3]. Among them some species are used as valuable Traditional Chinese Medicines (TCM). Phytochemical investigations on some *Ganoderma* species showed that ganoderma triterpenes (GTs) are mainly lanostanoid-type triterpenes with extensive biological and pharmacological activities, including cytotoxic [4,5], hepatoprotective [6,7], anti-inflammatory [8,9], and antioxidant [10] properties. *Ganoderma tropicum* is a main wild *Ganoderma* mushroom species found distributed in tropical areas of China. It is used as a health supplement and folk medicine alternative to *Ganoderma lucidum* and *Ganoderma sinensis* which are recorded in the Chinese Pharmacopeia to treat coronary heart disease and chronic hepatitis [11].

**Figure 1:**
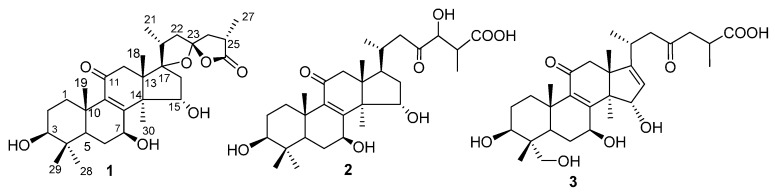
Structures of compounds **1**–**3**.

Acetylcholinesterase (AChE) inhibitors [12] have been considered promising tools to treat progressive degenerative neurologic disorders (Alzheimer disease and Parkinson's disease [13,14]). Previously, we have reported four new lanostanoids from the EtOAc extract of the fruit bodies of *G. tropicum*, among which 3β,7β,15β-trihydroxy-11,23-dioxolanost-8,16-dien-26-oic acid methyl ester showed AChE inhibitory activity [15,16]. In continuation of those studies, three new lanostanoids—ganotropic acid (**1**), 3β,7β,15α,24-tetrahydroxy-11,23-dioxolanost-8-en-26-oic acid (**2**) and 3β,7β,15α,28-tetrahydroxy-11,23-dioxolanost-8,16-dien-26-oic acid (**3**) (Figure 1) have been obtained from the *n*-BuOH extract of the fruit bodies of this fungus. In this paper, we describe the isolation, structural elucidation, and assay of AChE inhibitory activity of these new compounds.

## 2. Results and Discussion

**Figure 2:**
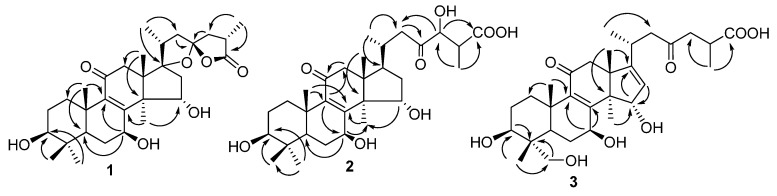
Selected HMBC (H→C) correlations of compounds **1**–**3**.

Compound **1** was obtained as a white power, and its molecular formula was assigned to be C_30_H_44_O_7_, with nine degrees of unsaturation, from its HREIMS (*m/z* 516.3095 [M]^+^). The IR spectrum revealed the presence of hydroxyl (3743 and 3442 cm^−1^), carbonyl (1768 cm^−1^) and double bond (1652 cm^−1^) absorptions. The ^1^H-NMR spectrum (Table 1) of compound **1** exhibited signals of seven methyls (δ_H_ 0.84 (3H, s, H-29), 1.01 (3H, d, *J* = 6.9 Hz, H-21), 1.02 (3H, s, H-28), 1.14 (3H, s, H-18), 1.24 (3H, s, H-19), 1.26 (3H, d, *J* = 7.2 Hz, H-27), 1.32 (3H, s, H-30)). The ^13^C-NMR and DEPT spectroscopic data (Table 2) showed 30 carbon resonances, including seven methyls, seven methylenes, six methines (three oxygenated), and ten quaternary carbons (two olefinic, two carbonyl, and two oxygenated), suggesting a triterpenoid skeleton. Further comprehensive analysis of the 1D and 2D NMR spectra indicated that compound **1** had the lanostane skeleton as ganoderic acid C_2_ [17]. However, the ^13^C-NMR data for compound **1** had two oxygenated quaternary carbons (C-23 and C-17) instead of the corresponding carbonyl (C-23) and methyl (C-17) in ganoderic acid C_2_. Apart from seven degrees of unsaturation (four rings, two carbonyls and one double bond), the remaining elements of the unsaturation in compound **1** were assumed to be two rings in the side chain. These were reminiscent of the presence of two oxygenic five-membered rings in compound **1** with the characteristic C-23 spiro carbon (δ_C_ 113.4) similar to that of abietospiran [18]. This partial structure was supported by HMBC correlations of H-27 with C-24 (δ_C_ 44.8) and C-26 (δ_C_ 179.2), H-24 and H-22 with C-23 as well as H-21 with C-17 (δ_C_ 94.9) and C-22 (δ_C_ 44.7) (Figure 2). The stereo-configuration of the lanostane skeleton for compound **1** was determined based on ROESY spectroscopic data and comparison of the NMR data with those similar structures. The NOE correlations of H-3/H-5, H-3/Me-28, H-7/H-5, H-7/Me-30 and H-15/Me-18 in the ROESY experiment indicated that 3-OH and 7-OH were β-oriented and 15-OH was α-oriented. 

**Table 1:** ^1^H-NMR (500 MHz) spectroscopic data (δ_H_ in ppm, *J* in Hz) of compounds **1** (in CDCl_3_) and **2**, **3** (in CD_3_OD).

H	1	2	3
1α	2.75 m	2.71 m	2.79 m
1β	0.92 m	0.94 m	0.97 m
2α	2.08 m	2.18 m	2.01 m
2β	1.64 m	1.63 m	1.67 m
3α	3.21 dd (*J* = 5.2, 11.0)	3.15 dd (*J* = 4.8,11.8)	3.60 dd (*J* = 5.0,11.8)
5α	0.89 d (*J* = 4.5)	0.95 d (*J* = 3.0)	1.40 d (*J* =12.5)
6α	2.10 m	2.30 m	2.03 m
6β			1.57 m
7α	4.59 dd (*J* = 7.2, 10.4)	4.53 dd (*J* = 7.4, 10.5)	4.56 dd (*J* = 7.3, 10.0)
12α	3.11 d (*J* = 15.6)	2.87 d (*J* = 15.2)	3.00 d (*J* = 14.4)
12β	2.25 d (*J* = 15.6)	2.41 d (*J* = 15.2)	2.75 d (*J* = 14.4)
15β	4.80 dd (*J* = 8.1, 8.6)	4.77 dd (*J* = 7.2, 9.3)	5.42 s
16α	2.40 dd (*J* = 8.1, 15.0)	1.81 m	5.25 s
16β	2.30 m		
17α	-	1.93 m	-
18	1.14 s	0.98 s	1.01 s
19	1.24 s	1.25 s	1.26 s
20	2.28 m	2.05 m	2.63 m
21	1.01 d (*J* = 6.9)	0.89 d (*J* = 6.4)	1.03 d (*J* = 6.8)
22	1.83 d (*J* = 14.0, α-H)	2.60 m	2.53 m
	2.70 dd (*J* = 6.7, 14.0, β-H)	2.50 m	2.30 m
24	2.04 m (β-H)	4.39 d (*J* = 5.0)	2.85 m
	2.48 dd (*J* = 8.1, 12.8, α-H)		
25	2.94 m	2.88 m	2.86 m
27	1.26 d (*J* = 7.2)	1.09 d (*J* = 7.1)	1.15 d (*J* = 7.0)
28	1.02 s	1.02 s	3.53 d (*J* = 11.2)
			3.31 d (*J* = 11.2)
29	0.84 s	0.84 s	0.72 s
30	1.32 s	1.26 s	1.28 s

**Table 2:** ^13^C-NMR (125 MHz) spectroscopic data (δ_C_ in ppm) of compounds **1** (in CDCl_3_) and **2**, **3** (in CD_3_OD).

C	1	2	3
1	34.7 (t)	35.9 (t)	35.4 (t)
2	27.9 (t)	28.8 (t)	28.0 (t)
3	78.4 (d)	73.2 (d)	72.4 (d)
4	38.8 (s)	39.7 (s)	43.3 (s)
5	49.1 (d)	50.4 (d)	52.9 (d)
6	27.7 (t)	29.0 (t)	28.5 (t)
7	69.2 (d)	70.1 (d)	69.7 (d)
8	158.8 (s)	161.3 (s)	162.3 (s)
9	141.9 (s)	143.5 (s)	142.7 (s)
10	38.7 (s)	39.7 (s)	39.8 (s)
11	200.2 (s)	202.3 (s)	201.9 (s)
12	47.2 (t)	53.2 (t)	48.5 (t)
13	49.7 (s)	48.4 (s)	53.0 (s)
14	54.5 (s)	55.3 (s)	57.6 (s)
15	73.2 (d)	78.9 (d)	78.1 (d)
16	45.0 (t)	37.1 (t)	125.6 (d)
17	94.9 (s)	49.0 (d)	155.9 (s)
18	19.9 (q)	19.8 (q)	20.7 (q)
19	19.5 (q)	17.5 (q)	20.0 (q)
20	44.0 (d)	33.3 (d)	28.6 (d)
21	18.3 (q)	20.2 (q)	22.7 (q)
22	44.7 (t)	46.7 (t)	49.5 (t)
23	113.4 (s)	212.9 (s)	209.9 (s)
24	44.8 (t)	78.9 (d)	47.4 (d)
25	35.7 (d)	43.2 (d)	35.9 (d)
26	179.2 (s)	177.6 (s)	179.6 (s)
27	15.1 (q)	11.4 (q)	17.5 (q)
28	28.3 (q)	28.7 (q)	66.2 (t)
29	15.8 (q)	16.4 (q)	13.0 (q)
30	21.4 (q)	20.0 (q)	23.3 (q)

The stereoconfigurations of chiral carbons C-17, C-20 and C-23 in the oxygenated five-membered ring in the side chain were determined to be the same as in abietospiran from their similar NMR data. The α-orientation of Me-27 was deduced based on analysis of ROESY correlations of H-24α (δ_H_ 2.48 dd (*J* = 8.1, 12.8)) with Me-21 and Me-27. Therefore, compound **1** was identified as (23*S*,25*R*)-3β,7β,15α-trihydroxy-11-oxo-17,23-epoxy lanost-8-en-26,23-olide, named ganotropic acid.

Compound **2** had a molecular formula C_30_H_46_O_8_ as established by its HREIMS (*m/z* 534.3202 [M]^+^), as well as its ^13^C NMR and DEPT spectroscopic data (Table 2) revealing 30 carbon resonances. Detailed analysis of its ^13^C-NMR spectra showed that compound **2** was highly similar to ganoderic acid C_2_, suggesting a lanostane skeleton. The only difference was the existence of the hydroxymethine signal δ_C_ 78.9 at C-24 in **2** instead of a methylene group in ganoderic acid C_2_. The hydroxymethine was assigned to be at C-24 based on the HMBC correlations of H-24 [δ_H_ 4.39 d (*J* = 5.0)] with C-26 (δ_C_ 177.6) and C-22 (δ_C_ 46.7). Compound **2** had the same basic lanostane configuration as that of ganoderic acid C_2_. The β-orientations of 3-OH and 7-OH were determined by ROESY correlations of H-3 [δ_H_ 3.15 dd (*J* = 4.8, 11.8)] with H-5, and H-7 [δ_H_ 4.53 dd (*J* = 7.4, 10.0)] with Me-28. The α-orientation of 15-OH was assigned from ROESY correlation of H-15 [δ_H_ 4.77 dd (*J* = 7.2, 9.3)] with Me-18. Based on above evidence, compound **2** was elucidated as 3β,7β,15α,24-tetrahydroxy-11,23-dioxo-lanost-8-en-26-oic acid.

Compound **3** was assigned the molecular formula C_30_H_44_O_8_ by analyses of its HREIMS (*m/z* 532.3030 [M]^+^) and ^13^C-NMR spectroscopic data revealing 30 carbon resonances. The ^1^H-NMR spectrum (Table 1) of compound **3** exhibited signals of six methyls and an olefinic proton (δ_H_ 5.25, s). Resemblance of its NMR spectroscopic data (Table 1 and Table 2) with those of 3β,7β,15β-trihydroxy-11,23-dioxolanost-8,16-dien-26-oic acid [16] suggested that their chemical structures were similar. The main difference was a methylol group at δ_C_ 66.2 (C-28) in compound **3** instead of a methyl in 3β,7β,15β-trihydroxy-11,23-dioxolanost-8,16-dien-26-oic acid, indicating that compound **3** was derived from the latter with an additional hydroxyl group attached to C-28. This was further confirmed by the HMBC correlation of H-29 (δ_H_ 0.72, s) with C-28. The stereo-configuration of compound **3** with a lanostane skeleton was confirmed by ROESY experiments. The β-orientations of 3-OH and 7-OH were determined by cross-peaks of H-3/H-5, H-7/H-5 and H-5/H-28 and the α-orientation of 15-OH was deduced by a H-15/H-18 cross-peak in the ROESY experiment. Accordingly, the structure of compound **3** was identified as 3β,7β,15α,28-tetrahydroxy-11,23-dioxo-lanost-8,16-dien-26-oic acid.

The inhibitory activities of compounds **1**–**3** against AChE were tested using a spectrophotometric method [19,20]. The results showed that these compounds possessed low percentage inhibition (<10%) at the concentration of 100 μM, compared to the tacrine control, indicating no significant inhibitory activities against AChE. In addition, these isolates were evaluated for antibacterial activity against *Staphylococcus aureus*, as well as cytotoxic activity against six tumour cells (K-562/HL-60/SMMC-7721/A-549/MCF-7/SW-480) according to the methods described previously [4,21], but they also showed no significant bioactivities.

## 3. Experimental Section

### 3.1. General Information

The NMR spectra were measured on an AV-500 spectrometer (Bruker, Bremen, Germany), using tetramethylsilane as internal standard. ESIMS spectra was recorded on an API Qstar Pulsar mass spectrometer (Bruker, Bremen, Germany) and HREIMS was measured with an Autospec Premier mass spectrometer (Waters, Milford, MA, USA). The IR spectra were obtained on a 380 FT-IR instrument (Thermo, Pittsburgh, PA, USA) using KBr pellets. UV spectra were measured on a UV-2550 spectrometer (Shimadzu, Kyoto, Japan). Optical rotations were recorded using a Autopol III polarimeter (Rudolph, Hackettstown, NJ, USA). Column chromatography (CC) was performed with silica gel (Marine Chemical Industry Factory, Qingdao, China), Sephadex LH-20 (Merck, Darmstadt, Germany) and RP-18 (Fuji Silysia Chemical Ltd, United States, 20–45 μm). TLC was performed with silica gel GF254 (Marine Chemical Industry Factory). Biological assay: ELISA Reader (ELx800, Bio-TeK, Winooski, VT, USA); Acetylthiocholine iodide, AChE, 5,5'-dithiobis-(2-nitrobenzoic acid) (DTNB) and tacrine (Aldrich 99%) were all bought from Sigma (Santa Clara, CA, USA). 

### 3.2. Fungal Material

Fruiting bodies of *G. tropicum* were collected in Lingshui County, Hainan Province, China (May, 2011), and identified by Prof. Xing-Liang Wu of Hainan University. A voucher specimen (No. 2011LZ01) is deposited at the Institute of Tropical Bioscience and Biotechnology, Chinese Academy of Tropical Agricultural Sciences.

### 3.3. Extraction and Isolation

Dried and powdered fruiting bodies of *G. tropicum* (6.5 kg) were extracted with 95% EtOH (3 L) at room temperature three times for 4 h each time. The combined extracts were concentrated and suspended in H_2_O followed by successive partitioning with EtOAc and *n*-BuOH, respectively. The *n*-BuOH extract (32.0 g) was separated by silica gel CC under reduced pressure using a solvent gradient of CHCl_3_-CH_3_OH (30:1→0:1, *v*/*v*) to afford five fractions (Fr1-Fr5). Fraction 4 (7.4 g) was separated by Rp-18 CC with MeOH-H_2_O (30:70→0:100, *v*/*v*) to give six subfractions 4a–4f. Subfraction 4a (1.6 g) was purified by silica gel CC eluted with PE-EtOAc (1:1) to obtain compound **1** (7.8 mg). Subfraction 4c (1.8 g) was repeatedly subjected to silica gel CC under reduced pressure eluted with CHCl_3_-EtOAc (1:1) and Sephadex LH-20 (CHCl_3_-MeOH 1:1) to yield **2** (8.0 mg) and **3** (4.2 mg).

### 3.4. Ganotropic Acid (**1**)

White power; ${\left[\alpha \right]}_{D}^{27}$ +20.0° (*c* 0.2, MeOH); UV (MeOH) λ_max_ (log ε) 254 (2.19), 213 (0.56), 204 (0.66); IR (KBr) *v*_max_ cm^−1^ 3743, 3442, 1768, 1652, 1514, 1461, 1389, 1056, 921; for ^1^H- and ^13^C-NMR spectroscopic data, see Table 1 and Table 2; positive ESI-MS *m/z* [M+Na]^+^ 539 (100); HREIMS (*m/z* 516.3095 [M]^+^, calcd. 516.3087 for C_30_H_44_O_7_).

### 3.5. 3β,7β,15α,24-Tetrahydroxy-11,23-dioxo-lanost-8-en-26-oic Acid (**2**)

Yellow oil; ${\left[\alpha \right]}_{D}^{27}$ +35.0° (*c* 0.2, MeOH); UV (MeOH) λ_max_ (log ε) 253 (2.24), 201 (2.69); IR (KBr) *v*_max_ cm^−1^ 3734, 2973, 1701, 1684, 1541, 1457, 1396, 1062, 992, 669; for ^1^H- and ^13^C-NMR spectroscopic data, see Table 1 and Table 2; positive ESI-MS *m/z* [M+Na]^+^ 557 (100); HREIMS (*m/z* 534.3202 [M]^+^, calcd. 534.3193 for C_30_H_46_O_8_).

### 3.6. 3β,7β,15α,28-Tetrahydroxy-11,23-dioxo-lanost-8,16-dien-26-oic Acid (**3**)

Yellow oil; ${\left[\alpha \right]}_{D}^{27}$ +15.0° (*c* 0.2, MeOH); UV (MeOH) λ_max_ (log ε) 255 (2.67), 205 (3.35); IR (KBr) *v*_max_ cm^−1^ 3730, 2926, 1734, 1652, 1541, 1457, 1396, 873, 669 ; for ^1^H and ^13^C NMR spectroscopic data, see Table 1 and Table 2; positive ESI-MS *m/z* [M+Na]^+^ 555 (100); HREIMS (*m/z* 532.3030 [M]^+^, calcd. 532.3036 for C_30_H_44_O_8_).

### 3.7. Bioassay of AChE Inhibitory Activity

AChE inhibitory activity of the three compounds was assayed by the spectrophotometric method developed by Ellman [19,20]. Acetylthiocholine iodide was used as substrate in the assay. Na_2_HPO_4_ (94.7 mL, 0.1 M) and NaH_2_PO_4_ (5.3 mL, 0.1 M) were mixed to prepare phosphate buffer (PB, pH 8.0). Compounds were dissolved in DMSO (2% in PB). The reaction mixture contained PB (110 μL), test compound solution (10 μL, 2000 μM) and acetyl cholinesterase solution (40 μL, 0.1 U/mL), which were mixed and incubated for 20 min (30 °C). The reaction was initiated by the addition of DTNB (20 μL, 6.25 mM) and acetylthiocholine iodide (20 μL, 6.25 mM). The hydrolysis of acetylthiocholine was monitored at 405 nm every 30 s. Tacrine was used as positive control. All reactions were performed in triplicate. The percentage inhibition was calculated as follows: % age inhibition = (E − S)/E × 100 (E is activity of the enzyme without test compound and S is the activity of enzyme with test compound).

## 4. Conclusions

The present study of the mushroom *G. tropicum* led to the isolation of three new lanostanoid triterpenes: ganotropic acid (**1**), 3β,7β,15α,24-tetrahydroxy-11,23-dioxo-lanost-8-en-26-oic acid (**2**) and 3β,7β,15α,28-tetrahydroxy-11,23-dioxo-lanost-8,16-dien-26-oic acid (**3**). Ganotropic acid possessed two oxygenic five-membered rings system in the side chain of lanostane skeleton. The evaluation on activities of these new compounds against AChE showed no significant inhibitory activity.

## Supplementary Materials

Supplementary materials can be accessed at: http://www.mdpi.com/1420-3049/20/02/3281/s1.
