# Supplementary material for: Three New Lanostanoids from the Mushroom Ganoderma tropicum
Source: Molecules. 2015 Feb 16;20(2):3281–9. doi: 10.3390/molecules20023281 (PMC6272550; doi:10.3390/molecules20023281)
Supplement: Supplementary file 1 [file molecules-20-03281-s001.pdf]

Supporting Information

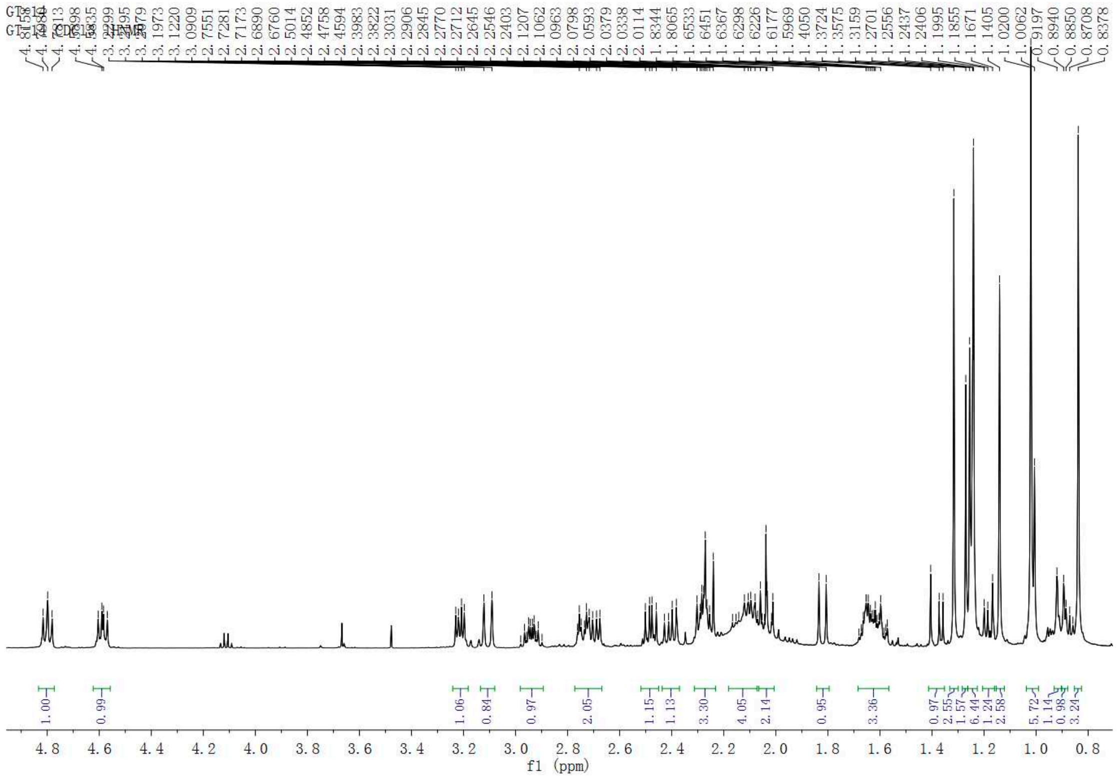

Figure S1. ^{1}H-NMR spectrum of compound **1** ($CDCl_3$, 500 MHz).

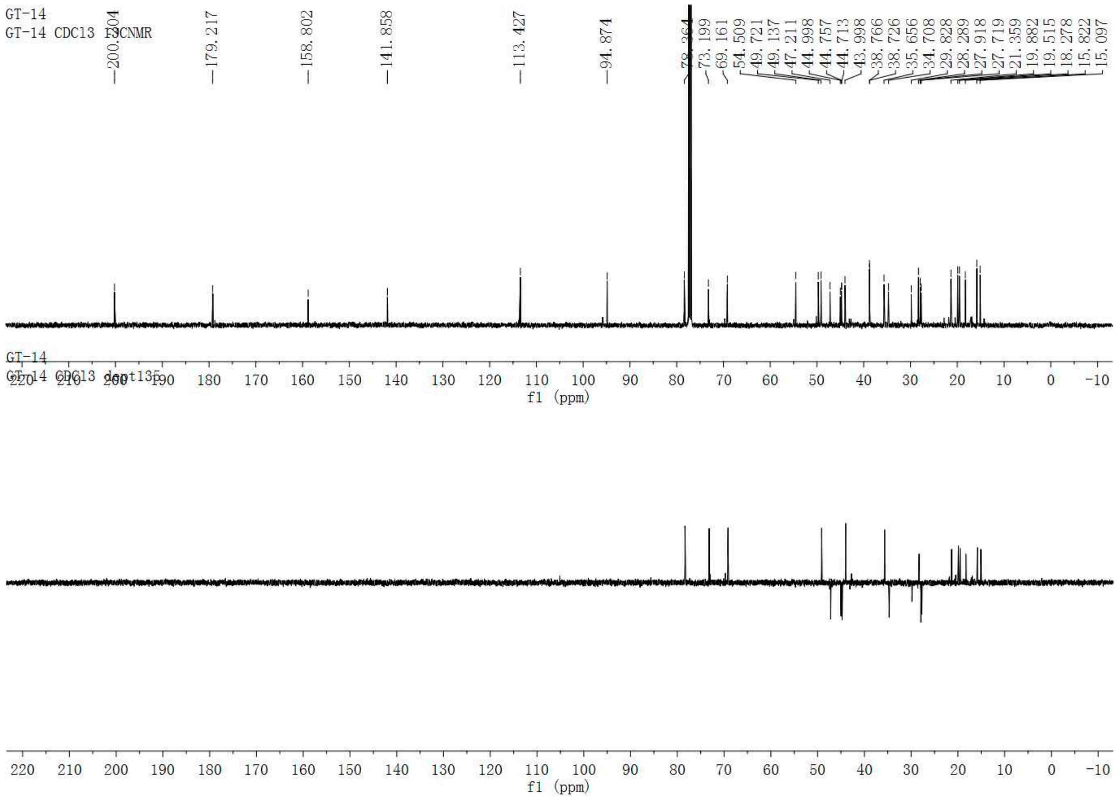

Figure S2. ^{13}C-NMR spectrum of compound **1** ($CDCl_3$, 125 MHz).

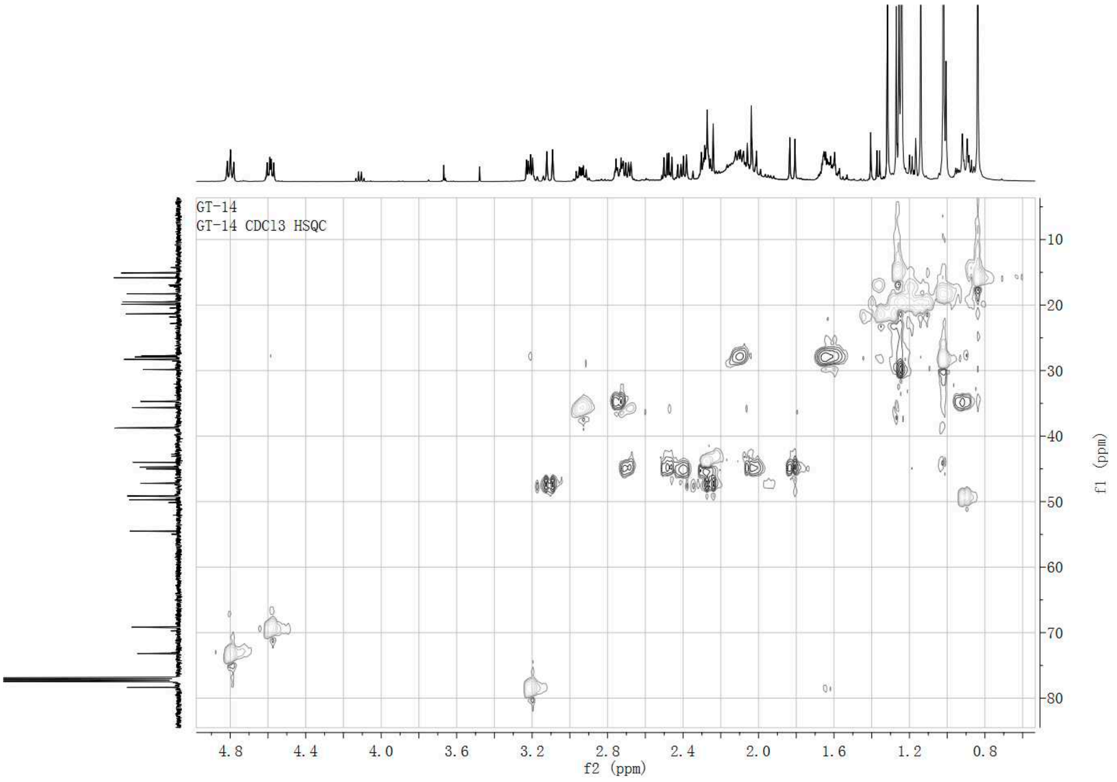

Figure S3. HSQC spectrum of compound **1**.

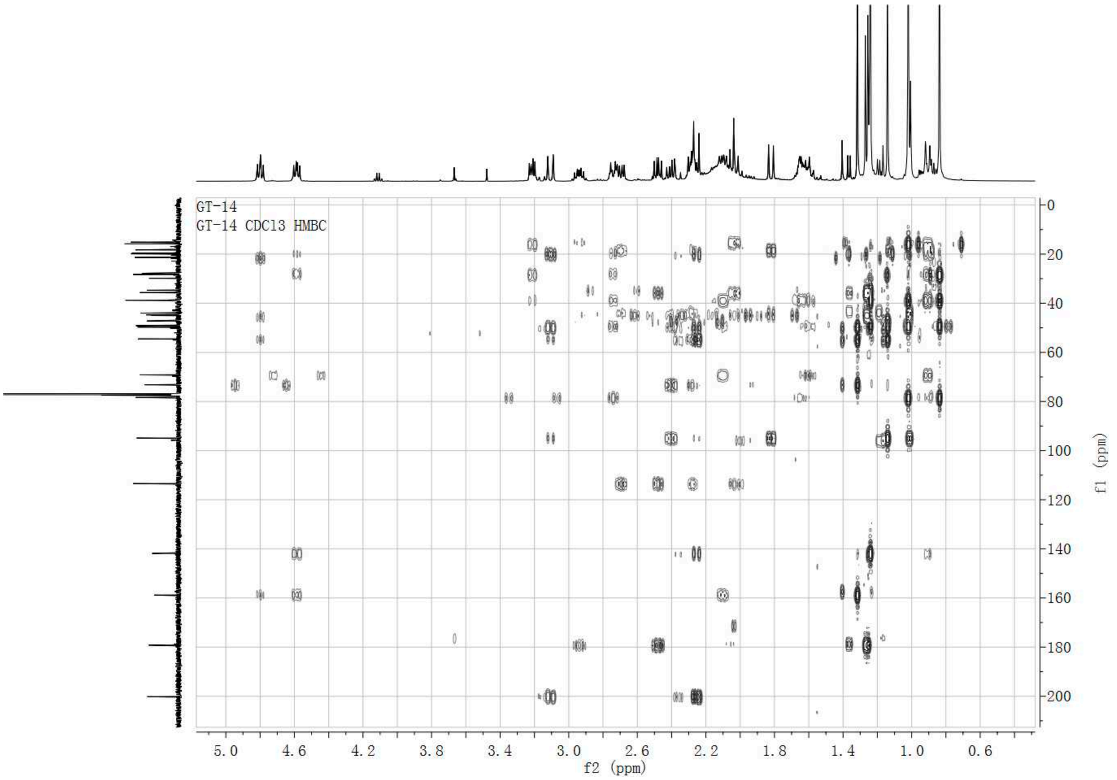

Figure S4. HMBC spectrum of compound **1**.

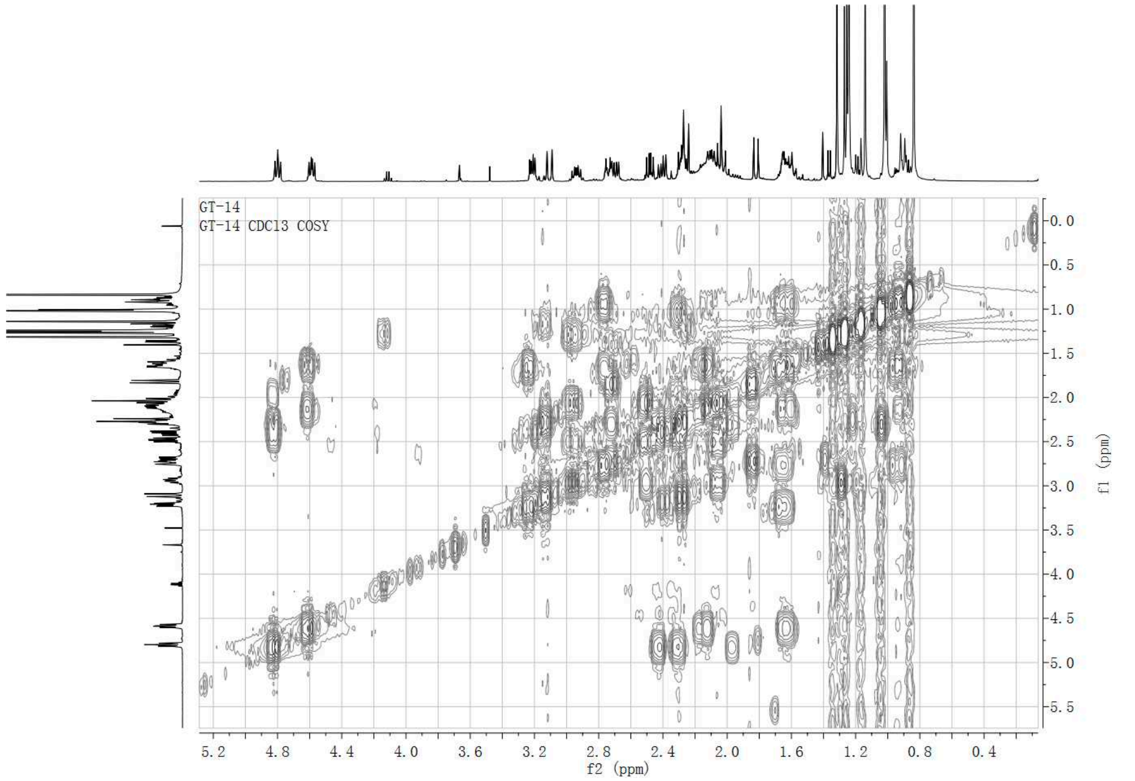

Figure S5. ^{1}H-^{1}H COSY spectrum of compound **1**.

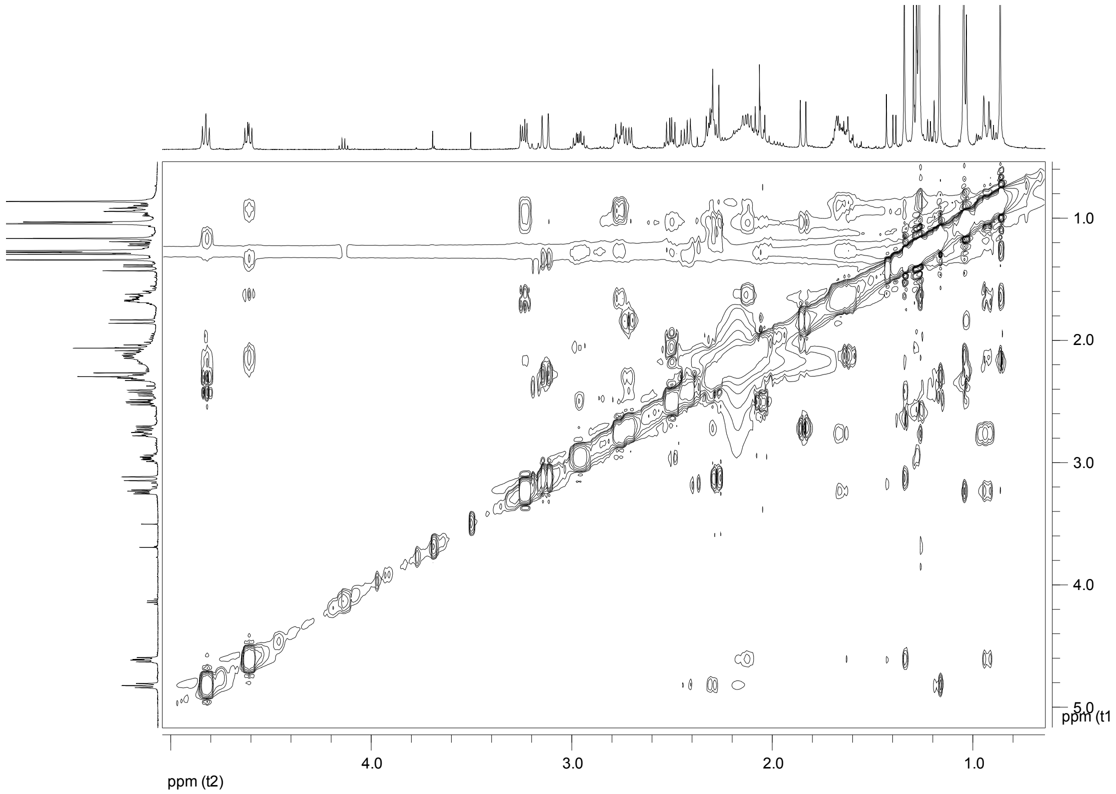

Figure S6. ROESY spectrum of compound **1**.
